# Changes in Drp1 Function and Mitochondrial Morphology Are Associated with the α-Synuclein Pathology in a Transgenic Mouse Model of Parkinson’s Disease

**DOI:** 10.3390/cells10040885

**Published:** 2021-04-13

**Authors:** Philipp Portz, Michael K. Lee

**Affiliations:** 1 Department of Medical Cell Biology, Institute for Anatomy and Cell Biology, University of Heidelberg, 69120 Heidelberg, Germany; philipp@portz.de; 2 Department of Neuroscience, University of Minnesota, Minneapolis, MN 55455, USA; 3 Institute for Translational Neuroscience, University of Minnesota, Minneapolis, MN 55455, USA

**Keywords:** Parkinson, mitochondria, fission, fusion, mitophagy, alpha-synuclein, Drp1, mitochondrial dynamics

## Abstract

Alterations in mitochondrial function and morphology are associated with many human diseases, including cancer and neurodegenerative diseases. Mitochondrial impairment is linked to Parkinson’s disease (PD) pathogenesis, and alterations in mitochondrial dynamics are seen in PD models. In particular, α-synuclein (αS) abnormalities are often associated with pathological changes to mitochondria. However, the relationship between αS pathology and mitochondrial dynamics remains poorly defined. Herein, we examined a mouse model of α-synucleinopathy for αS pathology-linked alterations in mitochondrial dynamics in vivo. We show that α-synucleinopathy in a transgenic (Tg) mouse model expressing familial PD-linked mutant A53T human αS (TgA53T) is associated with a decrease in Drp1 localization and activity in the mitochondria. In addition, we show that the loss of Drp1 function in the mitochondria is associated with two distinct phenotypes of enlarged neuronal mitochondria. Mitochondrial enlargement was only present in diseased animals and, apart from Drp1, other proteins involved in mitochondrial dynamics are unlikely to cause these changes, as their levels remained mostly unchanged. Further, the levels of Mfn1, a protein that facilitates mitochondrial fusion, was decreased nonspecifically with transgene expression. These results support the view that altered mitochondrial dynamics are a significant neuropathological factor in α-synucleinopathies.

## 1. Introduction

Parkinson’s disease (PD) is the second-most common neurodegenerative disease of the central nervous system, and affects about 1% of the population over the age of 60 [1]. PD is characterized by the presence of proteinaceous aggregates called Lewy bodies (LBs) and Lewy neurites (LNs) in brain tissue, and a severe loss of dopaminergic neurons in the substantia nigra pars compacta [2].

The current view is that abnormalities in α-synuclein (αS), the structural component of LBs and LNs [3], are mechanistically linked to neurodegeneration in PD. This view is supported by the fact that while a majority (~85%) of PD cases are considered idiopathic [1], missense mutations or gene multiplication in the SNCA gene, which encodes αS, cause highly penetrant, autosomal-dominant familial PD in humans [4]. Further, the expression of mutant human αS in transgenic (Tg) animals causes progressive neurodegeneration that is associated with αS aggregation [5,6]. The A53T Tg G2-3 mouse model (TgA53T^G2-3^) in particular provides a valuable tool to define the pathogenic mechanisms of PD and related α-synucleinopathies. TgA53T^G2-3^ mice exhibit progressive αS aggregation and neurodegeneration in the brainstem and spinal cord, leading to motoric dysfunction and premature mortality [5,6,7,8]. In addition to αS abnormalities, studies link PD with mitochondrial dysfunction [9,10,11]. Previously, we showed that α-synucleinopathy in the TgA53T^G2-3^ model was associated with mitochondrial dysfunction [6]. More recently, we showed that TgA53T^G2-3^ neurons exhibit defects in the actin dynamics, leading to reduced mitochondrial Drp1 and the elongation of the mitochondria [12]. Other studies have linked mitochondrial abnormalities with sporadic PD, including mitochondrial complex I deficits [9,13], mitochondrial DNA damage [14,15], and increase in oxidative stress [16]. Furthermore, various forms of hereditary PD are linked to genes encoded for mitochondrial quality control proteins such as Parkin [17] and Pink1 [18].

Mitochondria are highly dynamic organelles, existing in a constant equilibrium between fission and fusion. These processes govern mitochondrial dynamics and play a crucial role in the maintenance of their proper function e.g., by diluting damages to the mitochondrial population via fusion or enabling the disposal of damaged parts of the organelle through fission, autophagy, and mitophagy [19,20]. Major players in the regulation of these processes in the neurons are Dynamin-related protein 1 (Drp1) and Fis1 for fission, and Mitofusin 1 (Mfn1) and OPA1 for fusion of the outer and inner mitochondrial membranes, respectively. In recent years, studies of mitochondria have implicated impairments to mitochondrial fission/fusion and mitophagy in the pathogenesis of PD and other neurodegenerative diseases [12,21,22]. Thus, current results collectively support the view that mitochondrial dysfunction could be the common effector in PD, with a variety of causes. However, the structural basis for mitochondrial impairment in PD remains unresolved [12,21,23,24,25,26,27].

Given the pathological importance of αS pathology and mitochondrial abnormalities in PD, we sought to characterize possible defects in mitochondrial dynamics in the TgA53T^G2-3^ model of α-synucleinopathy. We show that α-synucleinopathy seems to selectively impact Drp1 localization to the mitochondria, as well as Drp1 phosphorylation as an indicator of reduced Guanosin-5′-Triphosphatase (GTPase) activity, leading to an increase in mitochondrial volume. Our results support the view that mitochondrial dysfunction is one of the downstream pathogenic effectors of αS pathology.

## 2. Materials and Methods

### 2.1. Animals

The generation of transgenic mice that express high levels of human mutant A53TαS under the control of a mouse prion protein promoter (TgA53T) has been described previously [5,6,7,8]. TgA53T^G2-3^ mice develop progressive neurological dysfunction at ~12 months of age, which rapidly progresses to end-stage paralysis within 14–21 days following the initial onset of symptoms. Age-matched non-transgenic (nTg) littermates were used as controls. All experimental protocols involving mice were in strict adherence to the NIH Animal Care and Guidelines and were approved by the Institutional Animal Care and Use Committee at the University of Minnesota. We used about an equal number of male and female mice in this study.

### 2.2. Subcellular Fractionation

In order to determine Parkin and Drp1 subcellular localization, we obtained enriched mitochondrial and cytosolic fractions from G2-3 mouse brain tissue [28] (Figure S2). Briefly, fresh mouse cortex (Ctx) and pooled brainstem/spinal cord (Br/SpC) tissue were homogenized in a 4 °C homogenization buffer (20 mM HEPS, 10 mM KCl, 1.5 mM MgCl_2_, 2 mM EDTA, 250 mM sucrose, 1× protease- and phosphatase-inhibitors; pH = 7.4) with a Teflon pestle. The homogenates were centrifuged at 1000 g, and the supernatant was then subjected to a 10,000 g spin to pellet the P10 fraction containing the mitochondrial fraction. The supernatant was collected and subject to a 100,000 g spin to pellet the microsomal P100 fraction. The supernatant from the 100,000 g spin was collected as the cytosolic fraction. The samples were constantly kept at 4 °C to avoid degradation.

### 2.3. Immunoblot Analysis

Immunoblot analyses of specific proteins separated by SDS-PAGE were performed as previously described [5,6,7,8]. Briefly, the protein concentrations were determined using a Pierce BCA Protein Assay Kit (Thermo Fisher Scientific Inc., Waltham, MA, USA) and equal amounts of total proteins in Laemmli buffer were separated by SDS-PAGE and transferred to a supported nitrocellular membrane (Bio-Rad Laboratories Inc., Hercules, CA, USA). The integrity of the proteins and proper transfer were confirmed via Ponceau-S staining followed by incubation in primary antibodies and HRP-conjugated secondary antibodies. To visualize the immunoreactive bands, the membranes were treated with SuperSignal West Pico Plus Chemiluminescent Substrate or SuperSignal West Femto Maximum Sensitivity Substrate (Thermo Fisher Scientific Inc., Waltham, MA, USA) and imaged using an ImageQuant LAS4000 (General Electric, Boston, MA, USA). The following primary antibodies were used: SantaCruz polyclonal rabbit anti-TOM20 #sc-11415 (1:1000), ThermoFisher Scientific monoclonal mouse anti-GAPDH #MA5-15738 (1:2000), Abcam monoclonal rabbit anti-Drp1 #ab184248 (1:1000), CellSignaling polyclonal rabbit anti-p(S616)Drp1 #3455 (1:1000), CellSignaling polyclonal rabbit anti-p(S637)Drp1 #4867 (1:1000), NovusBiologicals polyclonal rabbit anti-OPA1 #NB110-55290 (1:1000), Abcam monoclonal mouse anti-Mitofusin 1 #ab57602 (1:1000), ThermoFisher polyclonal rabbit anti-FIS1 #PA5-22142 (1:1000), CellSignaling polyclonal rabbit anti-H2A.X #2595S (1:1000), CellSignaling polyclonal rabbit anti-Transketolase #8616S (1:1000), Enzo polyclonal rabbit anti-Calnexin #ADI-SPA-860 (1:1000), ThermoFisher monoclonal mouse anti-ATP Synthase-α #459240 (1:1000), Abcam monoclonal rabbit anti-phosphorylated αS (phosphoSerine129; pS129αS) #ab51253 (1:1000), and BD Bioscience mouse monoclonal anti-αS #610787 (1:1000). The following secondary antibodies were used: ThermoFisher Goat IgG (H + L) Cross Adsorbed Secondary anti-mouse #G-21040 (1:10,000), ThermoFisher Goat IgG (H + L) Cross Adsorbed Secondary anti-rabbit #G-21234 (1:10,000).

### 2.4. Immunofluorescence Staining

Immunofluorescence staining was performed as previously described [29]. Paraformaldehyde-fixed brains were paraffin-embedded and sectioned into 5–6 µm thick slices. After deparaffinization and hydration, heat antigen retrieval was performed using BiocareMedical Reveal Decloaker in a vegetable steamer for 30 min. Subsequently, the specimens were washed with TBST (1× Tris Buffered Saline with 0.1% Tween), treated with 3% H_2_O_2_ for 10 min, and blocked with 100% BiocareMedical Background SNIPER blocking solution for 13 min. Primary antibodies were incubated over night at 4 °C in 5% SNIPER blocking solution with the following concentrations: SantaCruz polyclonal rabbit anti-TOM20 sc-11415 (1:200), WAKO monoclonal mouse anti-pS129 αS #015-25191 (1:300), Abcam monoclonal mouse anti-human αS *#27766* (1:200). The Abcam monoclonal rabbit anti-Drp1 #ab184247 (1:200) and Abcam monoclonal mouse anti-Cyclophilin F #ab110324 (1:200) antibodies were incubated over two days at 4 °C.

The secondary antibodies were incubated after the washing steps at 1:500 in 5% SNIPER blocking solution for 1 h at room temperature (ThermoFisher Scientific Goat anti-mouse Alexa Fluor 488 #A28175 and Goat anti-rabbit Alexa Fluor 555 #A27039). After three TBST washing steps, the slides were incubated with ThermoFisher Scientific DAPI (4′-6-diamidino-2-phenylindole) #D1306 1:500 in 5% SNIPER for 7 min. Finally, autofluorescence was quenched using a VectorLaboratories TrueView Autofluorescence quenching kit and the slides were mounted with the included VECTASHIELD Vibrance Antifade Mounting Medium.

### 2.5. Confocal Microscopy

3D z-stacks were acquired using an Olympus FluoView1000 confocal laser scanning microscope. Per animal, images were acquired for the TOM20-based morphological studies in two regions in the anterior (superior olivary complex) and posterior (medial vestibular nucleus) pons in the brainstem that display distinct phosphorylated alpha-synuclein staining and neurodegeneration in the Tg G2-3 mouse model, as well as in the somatosensoric cortex layer V. For the Cyclophilin-based Drp1 colocalization study, two 100× image stacks from both the posterior pons and the cortex of each animal were analyzed. For both studies, the matching regions were imaged in the non-Tg (nTg) control animals. The step size for z-stack acquisition was 0.42 µm, the exposure time was 20 µs per pixel and the aspect ratio was 1024 by 1024 for all images. The voltage and offset were set so that the strongest signal was just below the overexposure threshold.

### 2.6. Image Analysis

#### 2.6.1. Mitochondrial Morphometrics

Image stacks were analyzed using 3D reconstruction in Bitplane Imaris Software, and their semiquantitative Surface analysis tool, as previously described [30,31,32,33]. Briefly, neuronal nuclei were identified within 100× fields and a region of interest (ROI) was drawn around them. Within those ROIs, 3D reconstructions of the mitochondrial signal were created using the same reconstruction algorithms in both the Tg animals and the nTg controls. This enabled us to analyze large numbers of mitochondria, as each individual mitochondrial reconstruction provided a spatial extension of the designated mitochondrion in its spherical dimension. In order to blind this study, nuclei were picked with the channel of interest (mitochondrial marker and Drp1) not displayed. For the automated reconstruction of the mitochondrial signal, the algorithm used 0.1 µm for surface detail, the automated background subtraction used was 0.930 µm as the diameter of the largest sphere, automated thresholding and split touching objects were enabled with a 1.24 µm seed point diameter, and an automated quality threshold was used. For mitochondrial length and volume analyses in the cortex, about 80 mitochondria in 4 pSyn-positive neurons were analyzed per Tg animal, and about 140 mitochondria in 7 pSyn-negative neurons in the Tg and nTg animals. In the pons, about 280 mitochondria in 12 neurons per Tg and nTg animal were analyzed for both pSyn-positive and pSyn-negative neurons in both the posterior and the anterior regions. In total, we examined the following numbers of total mitochondria per group: cortex: nTg = 895, no pSyn = 1093, pSyn = 408; brainstem: nTg = 4076, no pSyn = 3044, pSyn = 3493.

For the mitochondrial 2D analysis, z-level projections of the confocal images were analyzed using the Mito-Morphology macro in FIJI (open-source ImageJ software) [34], as previously described [35]. Briefly, the mitochondrial signal was converted to grayscale, with color inversion and thresholding. Interconnectivity (area/perimeter), circularity, major axis length and object number were calculated with the “analyze particle” function.

#### 2.6.2. Distance Transformation

In order to analyze the average minimal distance of the Drp1 spots within the cell to the mitochondria stained with Cyclophilin F, eight neurons with the best immunostaining were chosen per region (cortex or brainstem) per animal. A 3D reconstruction of the Cyclophilin signal was created using the Imaris software as described above. The Drp1 signal was reconstructed in a cuboid ROI of 40 × 40 × 10 µm within each cell (picking was blinded by not displaying the channel of interest), using the Spot tool with an estimated xy diameter of 0.6 µm, background subtraction enabled, and the automated quality filter. Then, employing the Bitplane software, a distance transformation was created for the mitochondrial reconstruction (spots outside of surface), resulting in the creation of a channel that encoded the distance from the surface as channel intensity [36,37]. Thereby, by analyzing the minimal intensity of each Drp1 spot for the distance channel, the quantification of the average minimal distance between the mitochondria and Drp1 could be evaluated.

### 2.7. F-Actin/G-Actin Assay

The G-actin and F-actin assays were performed according to the protocol supplied in the Cytoskeleton Inc. G-Actin/F-Actin In Vivo Assay Kit #BK037. Briefly, fresh brain tissue was homogenized in F-actin stabilizing buffer containing protease- and phosphatase inhibitors and incubated at 37 °C for 10 min. Unbroken cells were then pelleted at 350 g. Afterwards, insoluble F-actin was pelleted at 100,000 g for 1 h at 37 °C so that the supernatant contained only G-actin. After carefully transferring the supernatant to a different tube, the pellet containing F-actin only, was resuspended in F-actin destabilizing buffer in order to drive its disassembly into G-actin.

Thereafter, samples were prepared in 1× Laemmli buffer and used for Western blot protein analysis using the polyclonal rabbit anti-actin antibody #AAN01 provided in the kit.

## 3. Results

### 3.1. Drp1 Translocation to Mitochondria Is Disrupted in G2-3 Mouse Brain

A previous study showed that mutant αS expression was associated with increased actin polymers and decreased mitochondrial Drp1 levels [12]. However, our biochemical analysis of polymerized actin did not reveal obvious alterations in the levels of actin polymerization in the TgA53T^G2-3^ mouse model (Figure S1). While we could not document increased actin filaments, we examined if the mutant αS-dependent decrease in mitochondrial Drp1 seen in flies [12] was also seen in TgA53T^G2-3^ mouse model. Drp1 (or DNM1L, DLP1, DVLP) is a GTPase of the Dynamin family that is mainly localized throughout the cytosol in the dense puncta, but is translocated to the mitochondrial outer membrane to promote mitochondrial fission [38,39]. Thus, the mitochondria-associated Drp1 levels are an indicator of mitochondrial fission activity.

**Figure 1:**
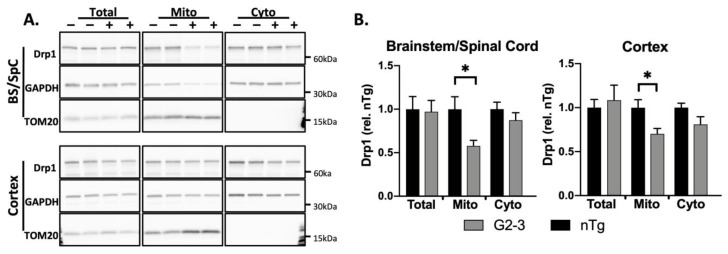
Drp1 translocation to the mitochondria is disrupted in TgA53T^G2-3^ mouse brains. (**A**) Brain subcellular fractions of nTg (−) and Tg (+) animals. (**B**) Quantitative analysis of Drp1 levels in the total, cytosolic (Cyto), and mitochondrial (Mito) fractions. The Drp1 levels were normalized to GAPDH or TOM20. Mitochondrial Drp1 levels are reduced by about 42% (*p* = 0.0318) in the BS/SpC, compared to about 30% decrease in the cortex (*p* = 0.0239). Multiple *t*-tests with Welch’s correction were performed (*n* = 6 per group). The values are mean ± SEM. (* *p* < 0.05).

We isolated crude mitochondrial and cytosolic fractions (Figure S2), as previously described [7], from endstage TgA53T^G2-3^ mice [5,40] and analyzed the Drp1 levels in the total homogenate, crude mitochondrial fraction, and cytosolic fraction. The enrichment of the mitochondria and cytosol was determined by evaluating TOM20 (translocase of outer membrane subunit 20) for the mitochondrial fraction (Mito), and examining GAPDH (glyceraldehyde 3-phopshate dehydrogenase) for the total and the cytosolic fraction (Cyto). The results indicated that the mitochondrial fractions from the brainstems and spinal cords (BS/SpC, pooled) of TgA53T^G2-3^ mice (+) contained reduced levels of Drp1, normalized to TOM20, compared to the levels in the BS/SpC from their nTg (−) litter mates (Figure 1A,B). The cytosolic levels of Drp1 were not different between the TgA53T^G2-3^ mice and their nTg littermates (Figure 1B).

Notably, the levels of Drp1 in the TgA53T^G2-3^ cortex were significantly lower than the levels in the nTg cortex. The TgA53T^G2-3^ cortex generally lacks severe αS pathology despite a high transgene expression. The results indicate that increased expression of the A53T-mutant αS in neurons is generally associated with reduced Drp1 association with the mitochondria.

**Figure 2:**
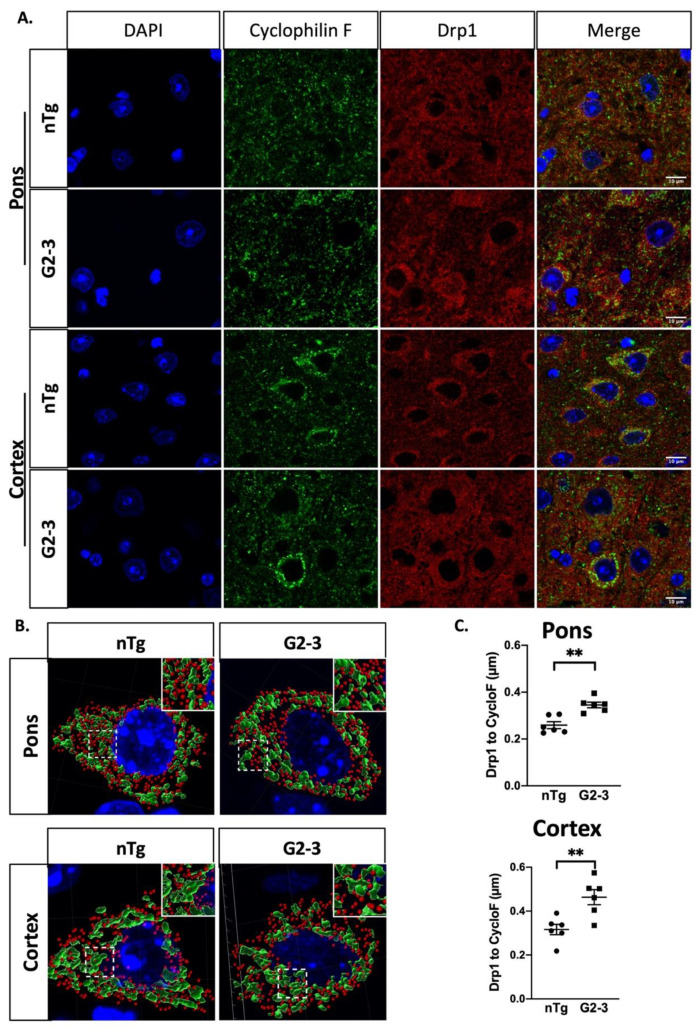
Mutant αS expression is associated with reduced Drp1 colocalization to mitochondria in vivo. (**A**) Immunofluorescent confocal imaging of mitochondria (Cyclophilin F) and Drp1 in mouse brains from nTg and TgA53TG2-3 (G2-3) mice. (**B**) Rendering of the 3D-reconstructed immunofluorescence signal in a representative neuron from the nTg (pons) and G2-3 (pons and cortex). (**C**) Quantifications of mean minimal distance between Drp1 and Cyclophilin F (Cyclo F) (mean ± SEM of means from each subject) show that in G2-3 mice, the mean minimal distance is increased by about 30%, and about 50% in the pons and cortex, respectively (*p* = 0.0013, *p* = 0.0066). An unpaired *t*-test with Welch’s correction (*n* = 6 per group) was performed. (** *p* < 0.01).

Previously, we showed reduced Drp1 localization in the mitochondria in the pontine neurons of endstage TgA53T^G2-3^ mice [12]. We extended our analysis by evaluating Drp1 localization with the mitochondria at the cellular level in both the cortical and pontine neurons. We performed an immunofluorescence staining of the Drp1 and the mitochondria (Cyclophilin F) (Figure 2A) using a z-level reconstruction of the confocal images (Figure 2B) obtained. By analyzing z-stacked images for mitochondrial (Cyclophilin F) and Drp1 signals using the MatLab Imaris software, we were able to approximate the mean minimal distance of each Drp1 signal to the closest mitochondrion, which appeared as punctate dots within a selected region of interest (ROI). The results (Figure 2C) show that the raw distances between Drp1 and the mitochondria were increased in both the pontine and cortical neurons of the TgA53T^G2-3^ mice (G2-3) compared to the neurons of nTg controls.

### 3.2. Phosphorylation Pattern of Drp1 in G2-3 Mics Suggests Mitochondrial Enlargement

Drp1 is a tightly regulated protein that can either be activated or inhibited with phosphorylation. Thus, its activity is determined not only by its localization on the mitochondria but also its phosphorylation dependent GTPase activity [41]. While phosphorylation at Ser616 is known to enhance Drp1-mediated fission [42,43], phosphorylation at Ser637 inhibits Drp1 activity [44,45,46]. Thus, we examined the phosphorylation of Drp1 at Ser616 and Ser637 in our samples.

**Figure 3:**
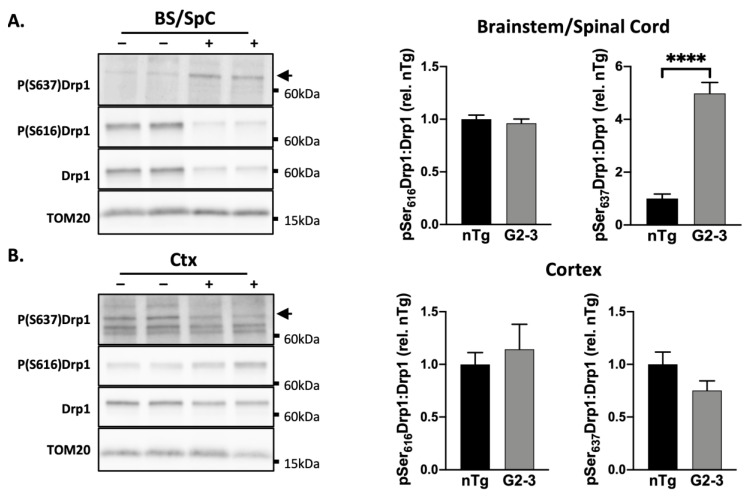
Phosphorylation pattern of Drp1 in brain areas with αS pathology is altered to favor reduced mitochondrial fission. (**A**,**B**) Immunoblot analysis of mitochondrial fractions from nTg control (−) and TgA53TG2-3 (+, G2-3) mice for phosphorylated Drp1. Location of the pSer637Drp1 band was identified using N2A cell lysate. Quantification of immunoblots with phosphorylated Drp1 normalized to the total mitochondrial Drp1. (**A**) In the pons, compared to the nTg samples, the G2-3 samples showed increased levels of pSer637Drp1 (*p* < 0.0001). (**B**) In the cortex, there is no change in pDrp1 levels compared to the nTg samples using an unpaired *t*-test with Welch’s correction (*n* = 6 per group, mean ± SEM). (**** *p* < 0.0001).

The mitochondria-enriched fractions were subjected to immunoblot analysis using two phospho-Drp1 antibodies, and correct band identification was confirmed using N2A total cell lysate with a prominent pDrp1 band (Figure S3). The results (Figure 3) show that the Drp1 phosphorylation states were obviously different in the BS/SpC of the TgA53T^G2-3^ subjects compared to those of nTg controls. While the level of pSer616Drp1, normalized to total mitochondrial Drp1 was not different between the TgA53T^G2-3^ and nTg subjects, there was a significant increase in the relative levels of pSer637Drp1 in the TgA53T^G2-3^ mice, compared to the nTg controls (Figure 3A). This pattern, combined with the reduced association of Drp1 with the mitochondria, would predict impaired mitochondrial fission in the BS/SpC. Unlike the mitochondrial Drp1 levels (Figure 1), the alterations in pDrp1 levels are correlated with the presence of αS pathology, as the relative levels of cortical pSer616Drp1 and pSer637Drp1 levels were not different between the TgA53T^G2-3^ and nTg subjects (Figure 3B).

Thus far, our results indicate that the increased expression of mutant αS reduces Drp1 localization on the mitochondria. However, the presence of α-synucleinopathy (the presence of αS aggregates indicated by insoluble αS and pS129αS staining [5,7,8,28]), may further exacerbate the loss of Drp1 function by reducing the activation state of Drp1.

### 3.3. Increased Mutant αS Expression Is Associated with Reduced MFN1 Levels

In addition to Drp1, the GTPases mitofusin 1 (MFN1) and dynamin-like 120 kDa protein (OPA1) are well-known regulators of mitochondrial dynamics. MFN1 and OPA1 mediate the fusion of the outer and inner mitochondrial membranes, respectively [47]. In addition, Fis1 is a cofactor for Drp1 [48] and/or a fusion inhibitor [49], and tips the equilibrium towards enhanced fission. Given the alterations in Drp1 levels in the TgA53T^G2-3^ models, we examined whether the expression of MFN1, OPA1, and Fis1 was altered as a function of α-synucleinopathy.

**Figure 4:**
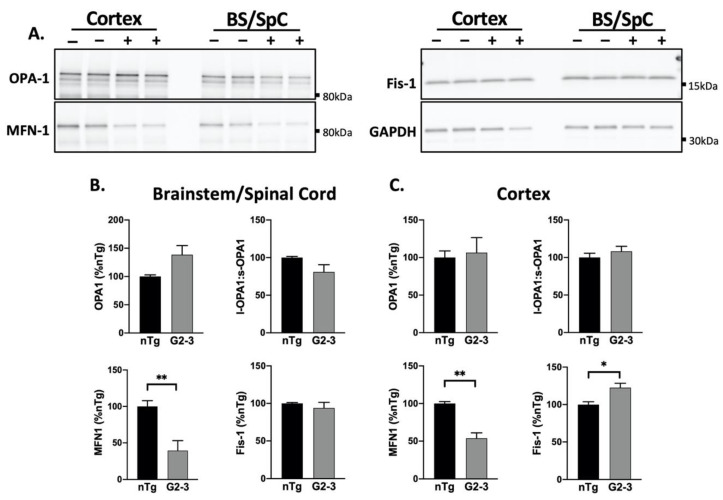
Decreased MFN1 levels with A53T mutant human αS expression in vivo. (**A**) Western blot of mitochondrial dynamics proteins in the total brain lysate of nTg (−) and Tg (+) animals. (**B**,**C**) A quantification of immunoblots shown in (**A**) normalized to GAPDH, show a decrease in MFN1 levels in the BS/SpC and the Ctx (*p* = 0.0074, *p* = 0018, respectively) of the TgA53TG2-3(G2-3) samples compared to the nTg samples. In the G2-3 cortex, Fis-1 levels were also increased (*p* = 0.0145), per the unpaired *t*-tests with Welch’s correction (*n* = 5 per group, mean ± SEM). (* *p* < 0.05, ** *p* < 0.01).

A previous report showed reduced MFN1 and MFN2 in the SpC of another TgA53T mouse model [27]. Our analyses show that the levels of MFN1 were significantly reduced in both the Ctx and BS/SpC of the TgA53T^G2-3^ subjects (Figure 4A–C). While we did not evaluate MFN2, based on a prior study we predict that it was also reduced.

The results also indicate that MFN1 levels were reduced by overexpression of A53T-mutant αS, irrespective of pathology, as the MFN1 levels were significantly reduced in both the Ctx and the BS/SpC. The OPA1 levels were comparable between the TgA53T^G2-3^ and nTg mice (Figure 4B,C). Moreover, while altered ratios between the long and short forms of OPA1 (L-OPA1:S-OPA1) is linked to impaired mitophagy [50] and decreased mitochondrial membrane potential [51], we did not observe alterations in the L-OPA1:S-OPA1 ratio. Finally, the Fis1 levels were also comparable between the Tg and nTg subjects in the BS/SpC, and were increased in the Ctx (Figure 4B,C).

Collectively, we propose that Drp1 is affected by mutant αS expression and pathology. The general loss of MFN1 in TgA53^G2-3^ neurons might be a compensatory response to counteract the loss of Drp1 function.

### 3.4. Increased Mitochondrial Volume and Length Are Associated with α-Synucleinopathy

The findings above indicate that both the expression of A53T-mutant αS and αS pathology could impact mitochondrial morphology. To determine how the reciprocal changes in the proteins involved in mitochondrial dynamics are reflected in mitochondrial morphology in presence or absence of αS pathology, we evaluated the overall volume and length of the mitochondria as a function of αS pathology. First, we confirmed that human αS was widely expressed by the transgenic neurons using an antibody that bound selectively to total human αSyn (hSyn) [52] (Figure S4). Consistent with previous findings, hSyn was widely expressed by neurons in both the pons and the cortex of the TgA53T^G2-3^ mice, but not in the nTg mice [5]. Furthermore, hSyn localization was consistent with enrichment at the presynaptic terminals, with a subset of the neurons showing strong somato-dendritic staining. The somato-dendritic hSyn immunoreactivity represents αS pathology because hSyn immunoreactivity is colocalized with anti-phosphoSer_129_αSyn (pSyn) staining (Figure S4). As previously described [5], based on the anti-pSyn staining, neurons containing αS pathologies were abundant in the pons, while only an occasional neuron exhibited αS pathology in the cortex.

**Figure 5:**
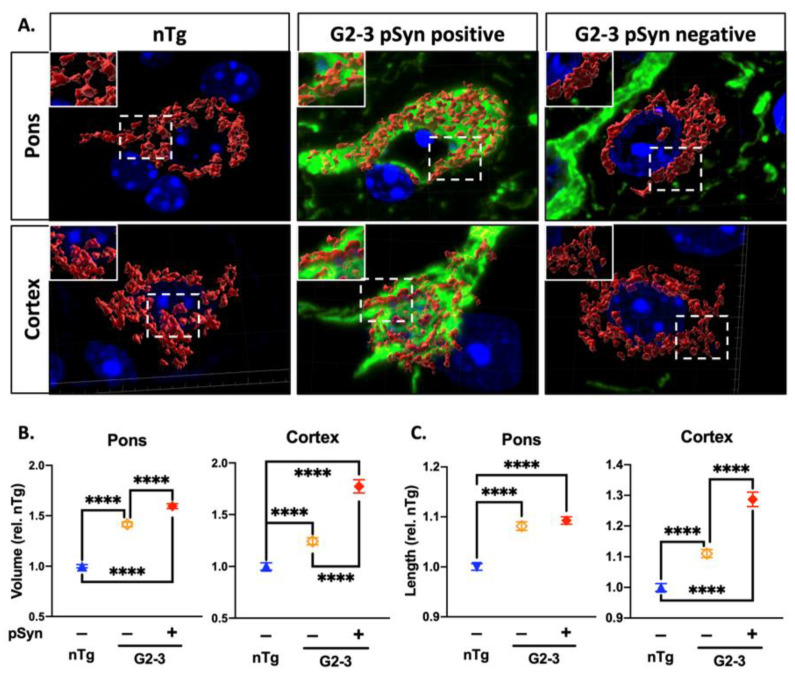
Increased mitochondrial volume and length is associated with αS pathology in TgA53TG2-3 Neurons. (**A**) 3D reconstruction of mitochondria (red) from z-level confocal images with DAPI (blue) and pSyn (green) fluorescence. (**B**) Morphometric quantification of mitochondrial volume and (**C**) length shows that mitochondrial volume and length are increased in pSyn-positive- and negative neurons, and that mitochondrial volume and length are differentially increased in the cortex in relation to pSyn immunoreactivity (*p* < 0.0001). Displayed are mean values ± SEM (SEM/SD). Volume pons: nTg = 0.01506/0.9615, no pSyn = 0.2385/1.316, pSyn = 0.02474/1.462; volume cortex: nTg = 0.03503/1.048, no pSyn = 0.03425/1.132, pSyn = 0.06435/1.3; length pons: nTg = 0.0066/0.4189, no pSyn = 0.0084/0.4661, pSyn = 0.0075/0.4453; length cortex: nTg = 0.0127/0.3799, no pSyn = 0.01318/0.4358, pSyn = 0.02361/0.4769), calculated using ordinary one-way ANOVA (*n* = 6 animals per group where at least 800 measurements were made per group). (**** *p* < 0.0001).

To determine mitochondrial morphology as a function of αS pathology, we performed a double-immunocytochemical analysis for αS pathology (pSyn) and mitochondria (TOM20) (Figure S5A). Then, we employed z-level confocal imaging to visually reconstruct the signal and render the 3D images of mouse mitochondria in vivo with high resolution (Figure 5A). By co-staining for pSyn, we were also able to examine mitochondrial morphology as a function of αS pathology. For neurons without αS pathology, we used nuclear morphology and the size of the soma to selectively identify them as neurons. 3D-reconstructed mitochondrial images (Figure 5A) were used to calculate the average neuronal mitochondrial volumes in the pons and cortex. In the pons and the cortex, the mean mitochondrial volume of the TgA53T^G2-3^ neurons was significantly higher than that of nTg neurons (Figure S5C). To better define the relationship between α-synucleinopathy and increased mitochondrial volume, we separated the mitochondrial volume based on the results of the pSyn staining. Our results show that in TgA535T neurons, both pSyn-positive and pSyn-negative neurons had larger mitochondria (Figure 5B) than did the nTg neurons. Significantly for TgA53T neurons, the mitochondrial volume in pSyn(+) neurons was significantly larger than in pSyn(−) neurons (Figure 5B). This result is consistent with the fact that αS pathology is associated with both reduced mitochondrial Drp1 (Figure 1 and Figure 2) and altered pDrp1 levels (Figure 3). Specifically, the loss of Drp1 function was exacerbated by αS pathology, leading to a further increase of mitochondrial volume in neurons exhibiting αS pathology.

In addition to the volume, both the elongation and lateral swelling of mitochondria has been associated with αS abnormalities [24,25,27,53]. To distinguish various contributors to increased mitochondrial volume in α-synucleinopathy, we examined the maximal mitochondrial internal length using our 3D-reconstructed images. Our results show that mitochondrial length was increased in the TgA53T^G2-3^ mice, as compared to the nTg mice (Figure S5C). When these data were grouped based on the presence or absence of pSyn, our results showed that all TgA53T neurons exhibited increased mitochondrial length (Figure 5C). In the cortex, the mitochondria of pSyn(+) neurons exhibited much more elongation compared to the nTg neurons, or neurons without a pSyn pathology (Figure 5C). However, while the mitochondrial length in the TgA53T^G2-3^ pontine neurons was longer than of the nTg neurons, there was no difference between pSyn(+) and pSyn(−) neurons (Figure 5C). The results indicate that in cortical neurons, αS pathology increases mitochondrial volume by increasing mitochondrial length. However, in the brainstem, because significant α-synu cleinopathy is associated with chronic inflammation, it is possible that both increased mitochondrial length and swelling contribute to the increased mitochondrial volume. One previous study [27] using another TgA53T model [54] concluded that mitochondrial fragmentation occurred in the TgA53T model based on a modest increase in the circularity of the mitochondria. While we did not see evidence of mitochondrial fragmentation, we used 2D analysis [27] to determine the indices of mitochondrial fragmentation (mitochondrial number per cell, circularity, interconnectivity, and length) in pontine neurons from nTg and TgA53T^G2-3^ mice. Our analysis failed to show significant differences in the number, circularity, or interconnectivity (Figure S6). However, consistent with our 3D analysis, we document a subtle increase in mean major axis length (Figure S6). Collectively, these results indicate that in our model of α-synucleinopathy, mitochondrial enlargement, rather than fragmentation, is the predominant mitochondrial phenotype.

### 3.5. Mitochondrial Enlargement Is Not Seen in Younger TgA53T^G2-3^ Mice

Thus far, our results indicate that mitochondrial abnormalities, particularly enlarged mitochondria, are seen in neurons exhibiting αS pathology. However, the fact that the mitochondrial volume was increased in both the pSyn-positive and pSyn-negative neurons raises the possibility that the mitochondria in tgA53T^G2-3^ mice were adversely affected by simple αS overexpression. To further address this issue, we examined mitochondrial morphology in young (3 and 6 months old) TgA53T^G2-3^ mice.

**Figure 6:**
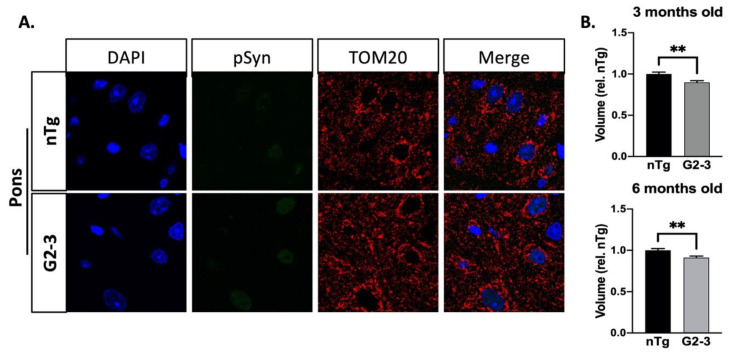
No mitochondrial enlargement is found in the pons of presymptomatic mice. (**A**) Representative confocal images of pSyn and mitochondria (TOM20) in three-month-old control and TgA53TG2-3 mice. Scale bar: 10 µm. (**B**) Quantification of mean mitochondrial and neuronal somas of 3- and 6-month-old mice shows mildly decreased mitochondrial volume by about 10% (3m.o.: *p* = 0.001; 6m.o.: *p* = 0.0019). SEM are displayed (SEM/SD: 3m.o.: nTg = 0.02267/0.7828, G2-3 = 0.02134/0.7483; 6m.o.: nTg = 0.02198/0.7703, G2-3 = 0.01825/0.6907), based on unpaired *t*-tests with Welch’s correction. (*n* = 3 per genotype). (** *p* ≤ 0.01)

Because TgA53T^G2-3^ animals develop pathology at ~10–12 months of age, they are pathology-free at 6 months of age [5]. An immunohistochemical analysis confirmed abundant hSyn expression in most neurons, comparable to that of the aged TgA53T^G2-3^ mice (Figure S7). At this age, pSyn accumulation was absent from the brainstem of the Tg mice (Figure S7). Analyses of mitochondrial morphology show that, at younger ages, neuronal mitochondria were not enlarged in the TgA53T^G2-3^ mice when compared to the nTg controls (Figure 6). In contrast, we saw a modest reduction in the mitochondrial volume of the TgA53T neurons. These results show that simple overexpression of mutant αS is not sufficient to increase mitochondrial volume, and that progressive mitochondrial enlargement and elongation occurs with both age and αS pathology. Moreover, a modest decrease in mitochondrial volume in younger Tg mice might reflect previous findings showing mitochondrial fragmentation with increased αS expression [24].

## 4. Discussion

In this study, we examined the TgA53T^G2-3^ mouse model of α-synucleinopathy for the relationship between αS pathology, proteins involved in mitochondrial fusion/fission, and mitochondrial morphology. Neurons rely on a healthy mitochondrial population, and mitochondrial dysfunction is associated with neuronal dysfunction/degeneration in vitro and in vivo [55,56,57,58,59]. Therefore, subtle changes in mitochondrial morphology and function are likely linked to detrimental effects on neuronal viability. Mitochondrial abnormalities have long been suggested to be a key driver of PD pathology. Genes linked to early-onset recessive PD are key regulators of mitochondrial quality control mechanisms [60], and exposure to mitochondrial toxins can also mimic PD in humans [60,61,62]. While numerous studies have linked αS abnormalities with mitochondrial dysfunction [6,63,64,65], very few have systematically examined how αS differentially impacts mitochondrial dynamics as a function of αS pathology. Our study extends previous results [12], largely derived from the αS fly model, and show that mutant αS expression and pathology in the mammalian brain are associated with reduced Drp1 function and mitochondrial enlargement. Specifically, we show that α-synucleinopathy is associated with reduced mitochondrial Drp1 (Figure 1 and Figure 2), increased levels of inhibitory pSer637Drp1, and decreased levels of fission-enhancing pSer616Drp1 (Figure 3). The alterations of pDrp1 were highly specific to the brainstem, a region that is more severely affected by αS pathology than the cortex. While we have not examined mitochondrial function, we previously showed that cytochrome c-oxidase 1 activity was reduced in the SpC without a decrease in the mitochondrial protein levels [6]. Furthermore, in flies, reduced mitochondrial Drp1 leads to increased mitochondrial volume and increased oxidation of mitochondria [12]. Thus, we propose that decreased Drp1 function and increased mitochondrial volume contributes to mitochondrial deficits in TgA53T^G2-3^ model.

Our results indicate that alterations to Drp1 levels are reflected in the mitochondrial enlargement in the neurons expressing mutant αS, which is exacerbated by the presence of αS pathology. This view is confirmed by the fact that in the cortex, mitochondrial enlargement was greater in the neurons harboring αS pathology (Figure 5). Additionally, while there was a global increase in the neuronal mitochondrial volume in endstage TgA53T^G2-3^ mice, younger Tg mice that had not developed αS pathology did not show increased mitochondrial volume in the pons (Figure 6). Thus, αS pathology affects mitochondrial morphology progressively. In addition, we previously showed that aged transgenic mice expressing wild-type human αS did not exhibit increased mitochondrial length [12], indicating a relationship between increased mitochondrial size and the pathogenicity of the αS transgene. In the pons, mitochondrial volumes in neurons without αS pathology might result from neuronal αS pathology that may be present in other parts of that neuron (axons and dendrites), which might have been overlooked by our analysis of αS pathology in the cell body, or the increased accumulation of toxic prefibrillar αS oligomers with aging. Furthermore, high levels of neuroinflammation and active neurodegeneration in the pons [5,6] could contribute to enlargement of the mitochondria in pSyn-negative neurons [66]. Because the cortical regions did not show increased inflammation or pDrp1 abnormalities, and generally had lower levels of αS pathology, mitochondrial elongation predominates in pSyn-positive cortical neurons (Figure 6B).

Previous studies that examined the effects of αS on mitochondrial morphology have mostly concluded that αS causes mitochondrial fragmentation [23,24,27,53,67]. In most of these studies, the forced overexpression of human αS, particularly the A53T mutant, led to the inhibition of mitochondrial fusion and/or mitochondrial fission/fragmentation. While our in vivo results indicate that neurons overexpressing A53T-mutant αS did not exhibit obvious signs of mitochondrial fragmentation, mutant αS overexpression in younger mice prior to onset of the pathology, was associated with a modest reduction in mitochondrial volume (Figure 6). However, with aging, and particularly with αS pathology, mitochondria became larger in vivo and do not show evidence of increased fragmentation.

It is notable that in cancer cells, increased mitochondrial activity is associated with increased Drp1 function, increased mitochondrial fragmentation, and decreased mitochondrial volume [68]. Cancer cells exhibit increased mitochondrial function to meet increased metabolic demand [69,70]. This is particularly true for large solid cancers, where cells survive in a hypoxic and low-glucose environment, like ovarian cancer [71]. Our results showing that the decreased Drp1 function associated with neurodegeneration is consistent with the known inverse correlation between the incidences of neurodegenerative disease and cancer [69], where neurodegenerative diseases are generally associated with decreased mitochondrial function [60,72]. The results from cancer cells support our view that reduced Drp1 function and increased mitochondrial size are associated with reduced mitochondrial function.

The proper regulation of the mitochondrial size via fission and fusion are important factors in maintaining a healthy mitochondrial population. Thus, the inhibition of fission and mitochondrial enlargement will likely contribute to the neurodegeneration associated with α-synucleinopathy [73,74]. Future research could extend our results using 3D electron microscopy and genetic manipulation of Drp1 expression. α-synucleinopathy is linked to altered kinase activities [8,75,76] and future studies could determine the relationship between kinase activation and Drp1 function in α-synucleinopathy. For example, Drp1 is phosphorylated at Ser-616 by MAPK1 and at Ser-637 by PKA [44,77], and our studies would predict that α-synucleinopathy is associated with increased PKA and decreased MAPK1 activation. Also, it will be important to define the functional implications of our findings in the context of senescence in cell and animal models treated with αS-preformed fibrils (PFF) [78].

In summary, our study examined the relationship between mutant αS expression, αS pathology, and mitochondrial morphology in vivo. We show that, unlike in cell culture models, mutant αS expression and pathology leads to Drp1 dysfunction and increased mitochondrial volume. Future studies may determine if restoring Drp1 function could represent a protective therapy for PD.

## Data Availability

Data available in a publicly accessible repository at doi:10.6084/m9.figshare.14397926.v1.

## Supplementary Materials

The following are available online at https://www.mdpi.com/article/10.3390/cells10040885/s1. Supplementary File 1 (Supplementary Figures; Figure S1: G-Actin/F-Actin Analysis in endstage Tga53T^G2-3^ mice; Figure S2: Western Blot of TgA53T^G2-3^ subcellular fractions for marker proteins H2A.X (nucleus), ATP-Synthase α (mitochondria), Calnexin (microsome), Transketolase (cytosol) shows sufficient enrichment of mitochondrial (P10) and cytosolic (Cyto) fractions; Figure S3: Western blot of TgA53T^G2-3^ total cortical lysate and N2A total cell lysate for p(Ser637)Drp1; Figure S4: Widespread transgene expression with abundant pSyn aggregation in the pons and the cortex; Figure S5: Confocal imaging of pSyn and mitochondria (TOM20) in control and TgA53T^G2-3^ mice and quantification of mitochondrial morphometrics; Figure S6: 2D Analysis of mitochondria in TgA53T^G2-3^ Pons from maximum intensity z-level projections of confocal images; Figure S7: Confocal imaging of hSyn immunoreactivity in 3 months old tgA53T^G2-3^ mice and age-matched controls shows hSyn staining in presymptomatic mice that is similar to endstage mice), Supplementary File 2 (Uncropped Gels/Western Blots).
