# Supplementary material for: Changes in Drp1 Function and Mitochondrial Morphology Are Associated with the α-Synuclein Pathology in a Transgenic Mouse Model of Parkinson’s Disease
Source: Cells. 2021 Apr 13;10(4):885. doi: 10.3390/cells10040885 (PMC8070398; doi:10.3390/cells10040885)
Supplement: Supplementary file 1 [file cells-10-00885-s001.pdf]

Supplementary File 1

Supplementary Figures S1-S7

for

Changes in Drp1 function and mitochondrial morphology are associated with the α-synuclein pathology in a transgenic mouse model of Parkinson's disease.

Philipp Portz and Michael K. Lee.

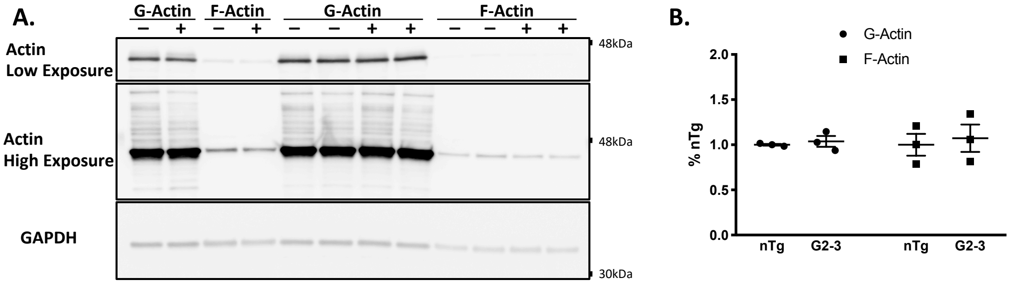

Supplementary Figure S1: G-Actin/F-Actin analysis of in endstage TgA53TG2-3 mice. (A) Western Blot for G-Actin after separation of filamentous and globular form. (B) Quantification of Western Blot shows no significant difference between nTg and TgA53TG2-3 mice. (n=3, mean±SEM)

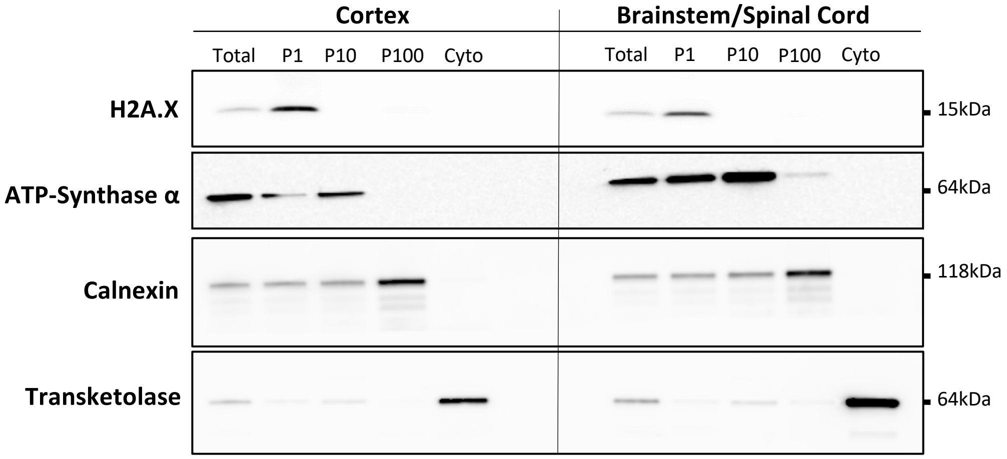

Supplementary Figure S2: Western Blot of TgA53TG2-3 subcellular fractions for marker proteins H2A.X (nucleus), ATP-Synthase α (mitochondria), Calnexin (microsome), Transketolase (cytosol) shows sufficient enrichment of mitochondrial (P10) and cytosolic (Cyto) fractions.

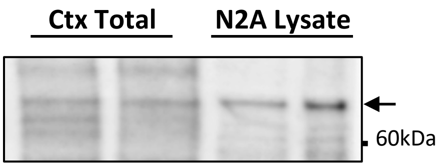

Supplementary Figure S3: Western blot of TgA53TG2-3 total cortical lysate and N2A total cell lysate for p(Ser637)Drp1. Identification of p(Ser537)Drp1 band was confirmed by comparing prominent p(Ser637)Drp1 band (arrow) in N2A lysate with the bands in cortical lysate on the same gel.

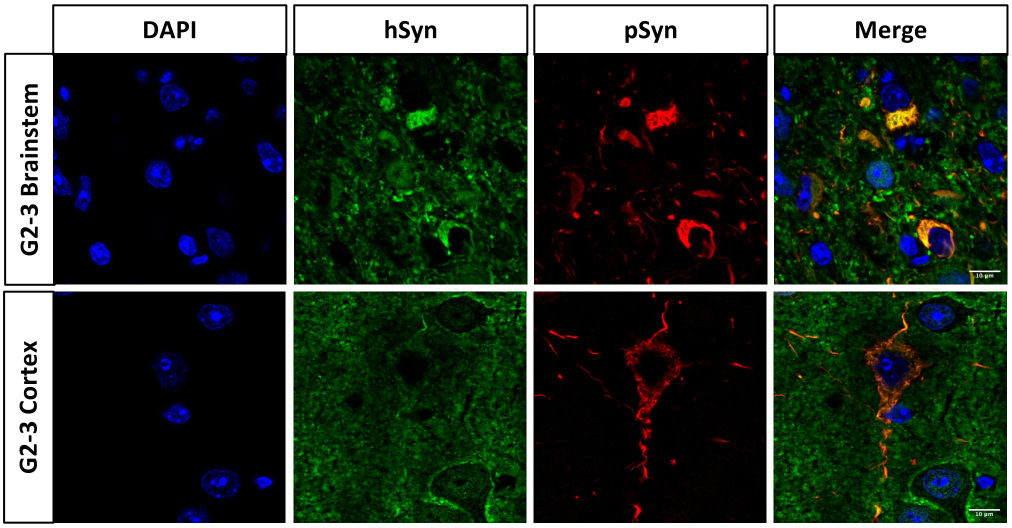

Supplementary Figure S4: Widespread transgene expression with abundant pSyn aggregation in the pons and the cortex. (A) Representative confocal images of pSyn and hSyn in pons and cortex of TgA53TG2-3 mice. Very few cortical neurons exhibit pSyn aggregation in spite of high transgene expression. Scale bar: 10µm.

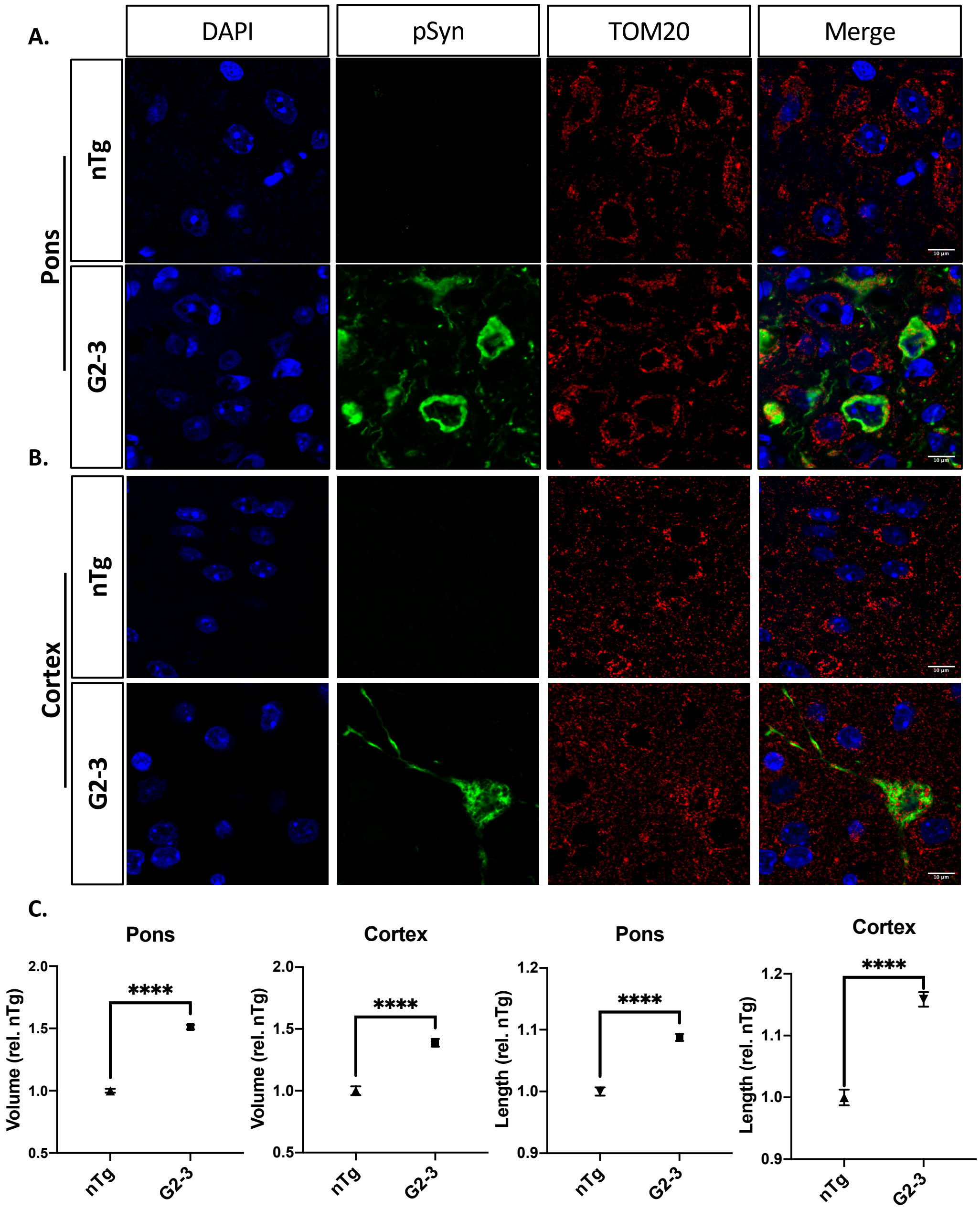

Supplementary Figure S5: Confocal imaging of pSyn and mitochondria (TOM20) in control and TgA53TG2-3 mice and quantification of mitochondrial morphometrics. (A, B) z-level maximum intensity projections of pontine and cortical TgA53TG2-3 neuronal mitochondria used for 3D reconstruction and morphometric analysis. (C) Quantification of mitochondrial morphometrics shows increased mitochondrial volume and length in tgA53TG2.-3 mice, when compared to nTg controls ($p<0.0001$), with cortical Tg length indicating greater elongation then pontine elongation. Unpaired t-test with Welch's Correction. Displayed are mean±SEM (SEM/SD: Volume pons: G2-3=0.0173/1.399; volume cortex: G2-3=0.03105/1.203; length pons: G2-3=0.0056/0.4551; length cortex: G2-3=0.01172/0.454) (n=6 animals per group). Scale bar: 10µm.

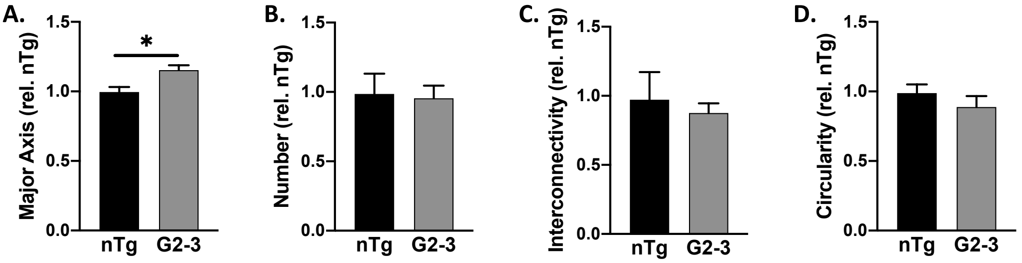

Supplementary Figure S6: 2D Analysis of mitochondria in TgA53TG2-3 Pons from maximum intensity z-level projections of confocal images: mean major axis length (A), mean somal number (B), mean interconnectivity (C) and mean circularity (D), relative to nTg. Increase in mean major axis length by about 15% (p=0.0118). Total of about 80 mitochondria analysed for TgA53TG2-3 nTg each. Unpaired t-test with Welch's Correction. (n=6 per group, mean±SEM)

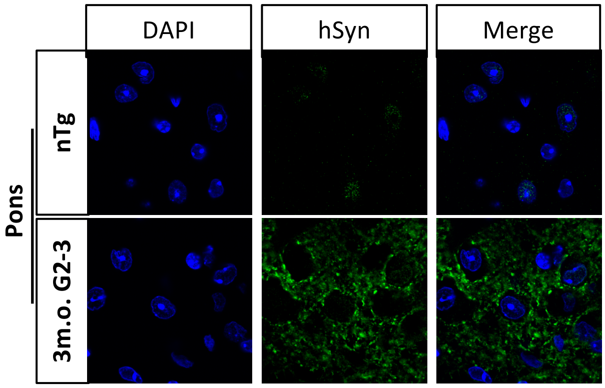

Supplementary Figure S7: Confocal imaging of hSyn immunoreactivity in 3 months old tgA53TG2-3 mice and age-matched controls shows hSyn staining in presymptomatic mice that is similar to endstage mice.
